# Gait improvement via rhythmic stimulation in Parkinson’s disease is linked to rhythmic skills

**DOI:** 10.1038/srep42005

**Published:** 2017-02-24

**Authors:** Simone Dalla Bella, Charles-Etienne Benoit, Nicolas Farrugia, Peter E. Keller, Hellmuth Obrig, Stefan Mainka, Sonja A. Kotz

**Affiliations:** 1 EuroMov, University of Montpellier, Montpellier, 34090, France; 2 WSFiZ in Warsaw, Department of Cognitive Psychology, Warsaw, 01-030, Poland; 3 Institut Universitaire de France (IUF), Paris, 75005, France; 4 Max Planck Institute for Human Cognitive and Brain Sciences, Department of Neuropsychology, Leipzig, 04103, Germany; 5 Télécom Bretagne, Lab-STICC, Brest, France; 6 MARCS Institute, Western Sydney University, Sidney, Australia; 7 Clinic for Cognitive Neurology, University Hospital and University Leipzig, Leipzig, 04103, Germany; 8 Neurologisches Fachkrankenhaus für Bewegungsstörungen / Parkinson, Beelitz-Heilstätten, 14547, Germany; 9 Faculty of Psychology and Neuroscience, Maastricht University, Dept. of Neuropsychology and Psychopharmacology, Maastricht, The Netherlands

## Abstract

Training based on rhythmic auditory stimulation (RAS) can improve gait in patients with idiopathic Parkinson’s disease (IPD). Patients typically walk faster and exhibit greater stride length after RAS. However, this effect is highly variable among patients, with some exhibiting little or no response to the intervention. These individual differences may depend on patients’ ability to synchronize their movements to a beat. To test this possibility, 14 IPD patients were submitted to RAS for four weeks, in which they walked to music with an embedded metronome. Before and after the training, patients’ synchronization was assessed with auditory paced hand tapping and walking to auditory cues. Patients increased gait speed and stride length in non-cued gait after training. However, individual differences were apparent as some patients showed a positive response to RAS and others, either no response, or a negative response. A positive response to RAS was predicted by the synchronization performance in hand tapping and gait tasks. More severe gait impairment, low synchronization variability, and a prompt response to a stimulation change foster a positive response to RAS training. Thus, sensorimotor timing skills underpinning the synchronization of steps to an auditory cue may allow predicting the success of RAS in IPD.

Gait and balance disorders are major therapeutic challenges in idiopathic Parkinson’s disease (IPD) as they strongly impact the activities of daily living, and are a growing economic burden for the health care system^1^. Gait deteriorates over time, impairing mobility, limiting independence, and reducing quality of life^2^. The increased likelihood of falls^3^,^4^ is a major reason for morbidity and disability in IPD^5^ leading to fractures and head injuries that may be fatal^6^. Unfortunately, gait and balance disorders respond poorly to long-term dopamine replacement therapy^1^,^7^. Therefore, additional non-pharmacological interventions to improve gait in IPD have been increasingly explored^8^.

Rhythmic auditory stimulation (RAS) has been shown to benefit IPD gait rehabilitation in several clinical studies^9^,^10^,^11^,^12^. The method uses external auditory stimulation to support gait initiation and continuation. Temporally predictable auditory cues have an immediate beneficial effect on gait by increasing speed, stride length, and improving symmetry and stability^13^. Benefits can generalize to non-cued gait after an extensive period of training with auditory cues^14^,^15^,^16^,^17^, resulting in increased mobility, enhanced quality of life, and a reduction of freezing episodes during movement^18^,^19^,^20^,^21^. Furthermore, there is evidence that musical cues can be more efficient than a simpler metronome stimulation, which is frequently used in research and clinical practice^22^,^23^. So far, RAS using musical stimuli has been evaluated only in a few randomized clinical trials^10^,^11^,^22^. In these studies the method was assessed based on the CONSORT Statement^24^ or using the PEDro scale^25^ (i.e., values >4). Evidence shows that training based on RAS with music or with simpler rhythmic stimuli is widely used, and generally effective in improving gait. However, there are indications that the success of this type of training varies significantly between individuals^12^. A better understanding of the causes of such variability may help to shed light on the functional mechanisms underlying the effects of RAS. Moreover, it may lead to individualized treatment and more efficient gait training in IPD.

An intriguing possibility is that the response to RAS is linked to the variability of patients’ sensorimotor timing skills. Distortions in timed perception and performance are often found in IPD^26^. In a variety of perceptual and motor timing tasks patients with IPD show a deficient performance^27^,^28^. For example, patients are more variable than controls in moving their finger to the pace indicated by a metronome^29^,^30^. Deficits can also be found in perceptual tasks, which need tracking the beat of rhythmic sequences^31^. However, there is considerable variability in IPD, with some studies showing spared motor and perceptual timing^32^,^33^. The ability to respond to rhythmic auditory cues may require relatively intact sensorimotor timing skills. In order to coordinate steps to the timing and rate of the auditory stimulation the patient must be able to extract the beat from an auditory sequence, such as a metronome or music, and to time goal-directed movements to the beat onsets. In particular, the extraction of a beat from a rhythmic auditory signal, the ability to match gait cadence to stimulus rate (i.e., the number of beats/minute), and the accuracy to synchronize heel strikes to the beat may be key factors for predicting the success of RAS. The neuronal network underlying sensorimotor timing, involving both subcortical and cortical regions such as the cerebellum, supplementary motor area, and premotor cortex has been associated with the beneficial effects of auditory cueing^10^,^34^,^35^. This network may support compensation of motor behavior in IPD patients via auditory cueing. This possibility is supported by the results of a PET study showing increased cerebellar (and parietal) activation after a cueing-based training programme^36^. Moreover, the importance of such general timing skills is consistent with recent evidence that the benefits of musically-cued gait training, a program based on RAS, extend beyond gait, and positively impact general perceptual and sensorimotor timing processes^37^. In sum, the variable outcome success of RAS across patients may depend on relatively spared individual sensorimotor timing capacities, a possibility, that has not been tested so far. Individual differences in sensorimotor skills may explain why auditory cueing is particularly effective for some patients but not for others. The goal of the current study is to test this link between the response to RAS and patients’ sensorimotor skills.

We examined the role of sensorimotor timing skills, measured with hand tapping and in gait as prime factors affecting patients’ response to RAS. To this end, IPD patients were submitted to a 1-month gait-training program using RAS with musical stimuli, and tested immediately after the training, and one month post-training. The rate of stimulation (i.e., beats/minute) was tailored to the patients’ preferred cadence (i.e., in the absence of stimulation). Note that immediate effects of RAS on gait are observed for a quite wide range of stimulation rates, from 90% to 125% of the preferred cadence leading to benefits on cadence^13^,^16^,^38^,^39^,^40^, stride length^13^,^16^,^40^,^41^, and gait velocity^16^,^38^,^39^,^40^,^41^. An optimal stimulation rate was selected for each patient (i.e., slower or faster than preferred cadence), as the one leading to the most visible effects on stride length, as a way to maximize the effect of the training. Patients’ ability to synchronize movements (steps and hand taps) to sounds was assessed before and after the gait training, and the results were then used to test whether sensorimotor timing skills were linked to the benefits of RAS on gait kinematics. We explored a variety of potential predictors of gait improvement supported by RAS using logistic regression models. The predictors were variables obtained from hand tapping and gait tasks, and from clinical tests. Variables reflecting sensorimotor timing skills, such as synchronization accuracy and variability, or the flexibility of the motor response to a stimulus change were expected to predict whether a patient responded positively or not to the training.

## Methods

### Participants

Twenty-one IPD patients were initially included in the study. Four patients were removed from further analysis, as they could not adhere to RAS training. Further, two patients, albeit initially included in the training group, dropped out at the beginning of the training sessions. Finally, one additional patient was excluded due to atypical gait performance during the training, and a concomitant cardiac disease. The final group consisted of 14 right-handed non-demented patients with IPD (5 females; mean age = 66.5 years, *SD* = 7.2), and corresponded to those patients, who were able to carry out the training program.

Diagnosis was established according to the UK PD Society Brain Bank clinical diagnostic criteria^42^. For the majority of the participants, the diagnosis was supported by a confirmatory nuclear medicine examination. Dementia was excluded using specific diagnostic clinical criteria^43^. Severity of motor symptoms was measured by the United Parkinson’s Disease Rating Scale (UPDRS, section III)^44^ and general severity of the disease was rated according to the Hoehn and Yahr (H&Y) scale^45^. Participants showed moderate symptoms of IPD with an average H&Y stage of 2 (*SD* = 0.7; range = 0.5–3) and UPDRS scores of 36.8 (*SD* = 19.1; range = 3–52). Patients exhibiting freezing of gait were excluded. Neuropsychological testing included the Token-test^46^, the Parkinson Neuropsychometric Dementia Assessment (PANDA)^47^ and the Consortium to Establish a Registry for Alzheimer’s Disease (CERAD)^48^ normed to an aged-matched group^49^. Inclusion criteria were mild to moderate motor symptoms, low scores (<5; *M* = 1.14) on the Geriatric Depression Scale^50^, normal hearing, and no severe additional neurological or psychiatric illness. All participants had little musical training (below 1 year), except one patient, who had 15 years of experience as an amateur musician. This patient, however, did not define herself as a musician, and her performance in gait and behavioral tasks did not stand out from the average of the other participants.

The levodopa equivalent daily dose (LED) was calculated both for dopamine replacement (L-Dopa LED) and for the dopamine agonists (Ago LED)^51^. Twenty right-handed, age-matched non-demented adults (females = 10; mean age = 66.4 years, *SD* = 7.8) formed the control group. Detailed demographic and clinical data for patients and controls are reported in Table 1. The methods were carried out in accordance with the approved guidelines. All experimental protocols and ethical consent forms were approved by the Ethics Committee of the University of Leipzig, Leipzig, Germany. Informed consent was obtained from all participants.

### Musically cued gait-training

The participants were trained using a program based on RAS, namely a musically-cued gait training (MCGT; see also Benoit *et al*.)^37^. The MCGT took place at the Clinic of Cognitive Neurology at the University Hospital of Leipzig. Patients were asked to walk along with a familiar German folk song (Hoch auf dem gelben Wagen). The patients did not receive any explicit instructions to synchronize their footsteps to the beat of the music. The song was presented without the lyrics, and the beat of the song was emphasized with a superimposed salient high-pitch bell sound. Optimal beat frequency was determined at the beginning of the first testing session. To create optimal stimulation conditions that guarantee maximal effectiveness of the MCGT, the beat frequency (i.e., beat rate of the music) of the rhythmic stimulation was individually adjusted for each patient in two respects. First, the stimulation was adapted to each patient’s preferred cadence (gait frequency). Each patient was asked to walk twice at her/his preferred cadence for 1 minute. Second, in two subsequent trials the patient was asked to walk for 1 minute at a beat frequency, which was either 10% slower or faster than her/his preferred cadence. The beat frequency (slower or faster), which led to the longest stride length was chosen and implemented in the MCGT for the entire duration of the training. Seven patients were trained with a beat frequency that was 10% faster than their preferred cadence, because in this condition they exhibited the greatest stride length (*M* = 1058.0 mm, *SEM* = 61.5; *W* = *−24, p* < 0.05). The other seven patients were trained with a beat frequency that was 10% slower than their preferred cadence, because this condition led to maximum stride length (*M* = 1052.4 mm, *SEM* = 64.7; *W* = −20, *p* = 0.05).

Each training session lasted 30 minutes and consisted of three phases. In the first and third phase (10 minutes) a patient’s gait was cued by music for 8 min. The stimulation was then stopped while the patient continued walking for 2 minutes at the same speed. In the second phase the patient performed stop-and-go trials. In the first 8 minutes of this phase the music was presented for 30 seconds. The end of the musical stimulus (after a short fade-out) signaled to the patient that s/he had to stop walking. After a 5-sec break, music was started again, and the patient was supposed to re-start walking. During the last 2 minutes of the second phase no music was played, and the patient was instructed to walk approximately for 30 sec, and then to stop and restart after waiting some time. It was up to the patient to choose when to start walking again, which typically took a few seconds. The goal was to train self-initiated gait in the absence of stimulation.

### Experimental protocol

Patients underwent 3 training sessions per week for one month (12 sessions overall). Medication was kept constant over the course of the study. Stimuli were delivered via a portable MP3-player and headphones (Sansa-Clip). Before MCGT, right after, and one month following the training, the patients were assessed in terms of a) gait performance (spatio-temporal gait parameters and step synchronization to auditory stimuli), and of b) sensorimotor timing skills in hand-tapping. Testing was spread over 2 days and performed at the Max Planck Institute for Human Cognitive and Brain Sciences, Leipzig, Germany. The tapping tasks were always performed before the gait tasks. The training and the assessment of patients’ performance was performed when patients were in full ON state (i.e., before the appearance of deleterious end-of-dose effects, such as dyskinesias). Controls’ performance was assessed only once.

#### Assessment of gait and of step synchronization to auditory stimuli

Gait kinematics were recorded with a Vicon MX Motion Capture System using Nexus software at a sampling frequency of 200 Hz. Small reflective markers (14 mm) were attached on the participants’ lower body in accordance with the Conventional Gait Model^52^. Participants walked following an oval trajectory in a dedicated motion capture room. In a non-cued condition participants walked for one minute at their preferred gait cadence. In two cued conditions, participants were asked to walk together with a musical stimulus with no explicit instruction to synchronize the steps to the beat. The stimulus was the same familiar song with a superimposed metronome used during the MCGT (i.e., samples of the folk song) in all 3 sessions (before, after, and 1 month after the training). The song was presented at two rates (one per condition), namely −10% and +10% relative to the participant’s preferred cadence. The non-cued condition was performed first, followed by the two cued conditions (+10% and −10%). The conditions were performed in this fixed order. For each condition there were 2 trials, one in which the participants walked clockwise, and the other when they walked counter-clockwise. The two trials were always performed in this order. Synchronization of audio and movement data was achieved by sending a trigger indicating the first beat of the audio signal to the motion capture system. This allowed to compute the degree of synchronization between the stimulus beats and the steps. Movement data were recorded and stored onto an IBM-compatible computer. The song was presented via wireless headphones (Beyerdynamic RSX 700). The tempo leading to the longest stride length was used for the MCGT.

#### Testing of sensorimotor timing with hand tapping tasks

Sensorimotor timing skills were assessed using the Battery for the Assessment of Auditory Sensorimotor and Timing Abilities (BAASTA)^37^,^53^. In the context of the current study, we report only two critical tasks, in which participants’ abilities to synchronize to an external predictable auditory stimulus, and to adapt to a stimulus change (i.e., an increase or a decrease of the stimulation rate). A full report of the results obtained with BAASTA for the same group of patients can be found in Benoit *et al*.^37^. These tasks involved hand-tapping^54^,^55^: paced tapping to an isochronous sequence, and an adaptive tapping task. In *paced tapping to an isochronous sequence*, the participants tapped with their hand to an isochronous sequence of 60 piano tones (tone frequency: 1319 Hz). There were three sequences, in which the inter-onset intervals (IOIs) between tones are 600, 450, and 750 ms. Each trial at a given tempo was repeated twice. In *adaptive tapping*, sequences of 10 tones were presented. The first 6 tones of a sequence had an IOI of 600 ms, while the remaining 4 tones either maintained the same IOI or, in 67% of the trials, were presented at a slower tempo (final IOI = 630 or 670 ms) or at a faster tempo (final IOI = 570 or 525 ms). The participants were asked to tap with their hand to the sequence of tones, to adapt to the tempo change, and to continue tapping at the new tempo after the presentation of the last tone for a duration corresponding to 10 IOIs. The task included 10 blocks, each formed by 6 trials (4 with tempo change, 2 without), presented in random order.

Hand tapping was performed on a Roland SPD-6 MIDI percussion pad controlled by MAX-MSP software (version 5.1) with 1-ms precision. Tapping data were recorded on an IBM-compatible computer, using the same timecode as that of the presented stimuli to allow the calculation of the synchronization between the stimuli and the taps. Stimuli were delivered over headphones (Sennheiser HD201) at a comfortable sound pressure level. No auditory feedback was provided during tapping. The tasks were preceded by practice trials.

### Data Analysis

Extraction of step and tapping times, and the analysis of gait and hand tapping data were carried out using dedicated Matlab routines. Data were pre-processed as follows. Events (steps or taps) leading to inter-event intervals smaller than 100 ms (artifacts) were rejected and outlier events were discarded. An outlier was defined as an event for which the interval between the actual step/tap and the preceding step/tap was smaller than Q1–3*Interquartile range (IQR) or greater than Q3 + 3*IQR, where Q1 is the first quartile and Q3 is the third quartile^53^. Moreover, tapping sequences obtained in the adaptive tapping task were rejected when participants were not able to synchronize with the metronome (i.e., with fewer than four taps produced in the second half of the synchronization phase corresponding to the pacing stimuli). A trial was treated as valid when it included eight taps without outliers in the continuation phase. Finally, a constant MIDI delay of 100 ms was subtracted from the hand tapping data.

#### Gait and step synchronization data

For the non-cued condition, standard spatio-temporal gait parameters were calculated using a previously described algorithm^56^, namely cadence (steps/minute), stride length of one cycle (mm), stride length variability (i.e., standard error of the mean - SEM - of stride lengths), gait speed (mm/second), stride time (seconds), stride time variability (SEM of stride times). For the cued gait conditions, three measures reflecting gait timing and synchronization of the heel strikes to the stimulus beat were calculated. We calculated the *inter*-*step interval* (in ms), namely the average duration between successive steps in a sequence of steps. Moreover, we computed synchronization accuracy and variability. *Synchronization accuracy* is the mean absolute asynchrony (i.e., not signed) between the step times and the musical beats. Small asynchrony indicates high accuracy. *Synchronization variability* is the SEM of asynchrony between the step times and musical beats. Both synchronization accuracy and variability are indicated in percent of the IOI. Similar measures of timing and synchronization are used below to quantify the performance in synchronized hand tapping tasks.

#### Hand tapping data

In paced tapping to an isochronous sequence, tapping sequences were analyzed to obtain three measures reflecting tap timing and synchronization of the taps to the stimulus beat. The *inter*-*tap interval* (in ms), namely the average duration between successive taps in a sequence, was calculated. In addition, synchronization accuracy and variability were computed. *Synchronization accuracy* (in % of the beat IOI) is the mean absolute asynchrony (i.e., not signed) between the tap times and the onset of the metronome tones. *Synchronization variability* (in % of the beat IOI) is indicated by the SEM of asynchrony between the tap times and the metronome tones. In this task, the results obtained in the trial showing the lowest variability were submitted to further analyses.

In the adaptive tapping task, adaptation of tapping to the tempo change was measured by calculating the adaptation index. This index is computed based on the taps obtained in the continuation phase, as done in Schwartze *et al*.^57^. Mean inter-tap intervals in this phase were considered as a function of the final sequence tempo, and regression lines were fitted to the slopes of these functions. The slopes were used as adaptation indices. An Adaptation index of 1 indicates perfect adaptation; values lower than 1 indicate undercorrection, and values greater than 1, overcorrection. In addition, error correction was computed and partitioned into phase correction (“phase” throughout the manuscript, for simplicity) and period correction (“period”) based on the fit of the data to the two-process model of error correction^57^,^58^. Phase indicates the change in phase of the impulses (or oscillations) of an internal clock without changing its frequency or period. Period refers to a change in the frequency of the internal clock in order to adapt to the frequency of the external pacing stimulus. Phase and period were estimated by determining the values of the parameters that led to the best fit between predictions based on the two-process model of error correction (implemented in MATLAB), and the observed adaptation indices^59^. The adaptation index, phase, and period were obtained separately for tempo acceleration (i.e., faster tempi with final sequence IOIs <600 ms) and tempo deceleration (slower tempi with final sequence IOIs >600 ms).

## Results

Spatio-temporal gait parameters in the non-cued condition for controls and IPD patients before and after MCGT are reported in Table 2. Some data, namely the effect of RAS on perceptual timing and sensorimotor tasks, have been previously published elsewhere (Benoit *et al*.)^37^. Non-parametric statistics (Mann-Whitney tests for comparing patients to controls, Wilcoxon tests for within-subject comparisons) were used whenever data were not normally distributed according to Kolmogorov–Smirnov tests. Compared to controls, patients exhibited faster cadence (*U* = 86, *p* < 0.05), shorter stride length (*U* = 76, *p* < 0.05), shorter stride time (*U* = 83, *p* < 0.05) and marginally slower speed (*U* = 102, *p* = 0.09) prior to the training. Gait speed (*W* = −51, *p* < 0.05) and stride length (*W* = −55, *p* < 0.05) increased significantly after the training. This effect was still significant one month after MCGT (speed, *W* = −67, *p* < 0.05; stride length, *W* = −63, *p* < 0.05). Notably, the patients’ gait speed improved to the level of controls at post-training and at follow-up, while stride time was significantly shorter both at post-training (*W* = 55, *p* < 0.05) and at follow-up (*W* = 71, *p* < 0.05) as compared to pre-training. Patients’ variability in stride time was significantly reduced immediately after the training (*W* = 65, *p* < 0.05), however, this effect was not maintained at follow-up (*W* = 37, *p* = 0.13).

Synchronization performance in the two cued gait conditions (10% slower and faster that the preferred cadence), namely mean inter-step interval and synchronization accuracy and variability are also reported in Table 2. As can be seen, IPD patients on average were able to increase or decrease their cadence as a function of the stimulus rate. Indeed, IPD patients did not differ from controls in terms of inter-step interval prior to the training (−10% of preferred cadence, *U* = 106, *p* = 0.26; +10%, *U* = 92, *p* = 0.19). MCGT affected their inter-step interval. In general the patients showed shorter inter-step intervals after MCGT, an effect that is visible when they walked with a slower beat rate (post-test, *W* = 57, *p* < 0.05; follow-up, *W* = 75, *p* < 0.01) than with a faster stimulus rate (only follow-up, *W* = 48, *p* < 0.05). This finding is consistent with the observation that the patients overall increased their gait speed after MCGT. No major differences were found at the group level between patients and controls in terms of synchronization accuracy and variability. Although in general patients seem to be less accurate and more variable than controls before the training, this difference reached statistical significance only in one case (accuracy, beat rate = −10%, *U* = 48, *p* < 0.01). After MCGT, synchronization variability increased only when patients walked at a faster beat rate (post-test, *W* = −50, *p* < 0.05; follow-up, *W* = −62, *p* < 0.01). To sum up, at a group level, in spite of a visible and consistent effect of MCGT on non-cued gait, this is not clearly accompanied by improved synchronization of footfalls to the beat of music. However, this finding does not preclude that step synchronization to the beat may play a significant role in explaining individual differences among patients. This possibility is examined later on.

Performance in hand tapping tasks did not differ significantly in IPD patients and controls before the training. The only effects of MCGT on tapping performance were a reduction of synchronization variability with an IOI of 600 ms (pre-test, *M* = 0.52, *SEM* = 0.02; follow-up, *M* = 0.47, *SEM* = 0.03; *W* = 67, *p* < 0.05), and greater accuracy at an IOI of 750 ms (pre-test, *M* = 6.3, *SEM* = 0.04; follow-up, *M* = 5.8, *SEM* = 0.06; *W* = 63, *p* < 0.05). In addition, period correction was greater in the adaptive tapping task for deceleration only (pre-test, *M* = 0.78, *SEM* = .10; follow-up, *M* = 1.10, *SEM* = 0.10; *W* = −48, *p* = 0.05) (for details see Supplementary Table 1).

### Individual differences

**Figure 1:**
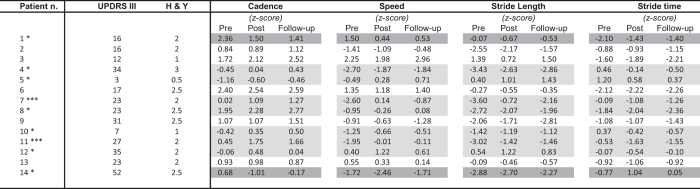
Individual gait performances in non-cued gait pre-, post-training, and at the follow-up in IPD patients. Gray shading indicates patients showing significant differences in gait speed between pre- and post-treatment according to Hass *et al*.^60^ criteria. ^*^Small effect. ^**^Average effect. ^***^Large effect.

Analyses were performed to quantify the benefits of MCGT at the individual level. Figure 1 shows individual performance scores for non-cued gait in the 14 IPD patients before and after the training. The results are expressed in *z*-scores relative to the performance of controls. Significant differences between groups are highlighted (gray shading). Patients’ response to MCGT was qualified using clinically meaningful criteria in terms of gait speed as indicated by Hass and collaborators for IPD^60^. Differences in gait speed between pre- and post-treatment were defined based on distribution-based analyses and effect size metrics. A small difference in gait speed was 06 m/s, a moderate difference was 0.14 m/s, and a large difference was 0.22 m/s. In the Figure, patients showing positive and negative differences (i.e., positive and negative effects of MCGT) based on these criteria were indicated with a light gray and dark gray shading, respectively. Stars indicate whether the effect is small, average, or large. Half of the patients benefitted from MCGT: 5 of them showed a small effect, and 2 a large improvement in gait speed. Among the remaining 7 patients, 5 showed no effect, which cannot be qualified clinically as positive or negative, while 2 exhibited worsened performance after MCGT.

In order to shed light on the factors that are linked to this variability in the response to RAS, further analyses were conducted using logistic regression modeling. Patients were divided into two categories based on their response to MCGT, according to Hass *et al*’s criteria^60^. One category, referred to as “positive response” included all the patients who showed at least a small increase in speed after the training (*n* = 7). The other category (“no positive response”) included the remaining patients who either did not respond to the training or whose performance slightly worsened (*n* = 7). Patients’ category (no positive response vs. positive response to MCGT) was entered as a binary dependent variable (0/1) into a logistic regression model. Different models were tested using predictors issued from both gait performance and hand tapping tasks. An optimal model, leading to the most satisfactory fit to the data was selected to be presented in this paper. The following variables, obtained for patients at each time of testing, were entered as continuous predictors into the model: gait speed, synchronization accuracy (gait, condition +10%), synchronization accuracy (paced tapping, average across tempos), synchronization variability (paced tapping, averaged across tempos), adaptation index (adaptive tapping, for acceleration), and phase correction (adaptive tapping, for acceleration). Time of testing (pre vs. post vs. follow-up) was also initially included as a categorical predictor in the model together with the aforementioned predictor variables. However, because the contribution of this predictor to the model was not significant, and considering that this variable did not significantly improve the model fit, time of testing was removed from the final model. The possibility that other clinical variables, such as the UPDRS and H&Y scores could explain different responses to MCGT was also examined. None of these variables contributed significantly, thus they were not entered into the final model.

**Figure 2:**
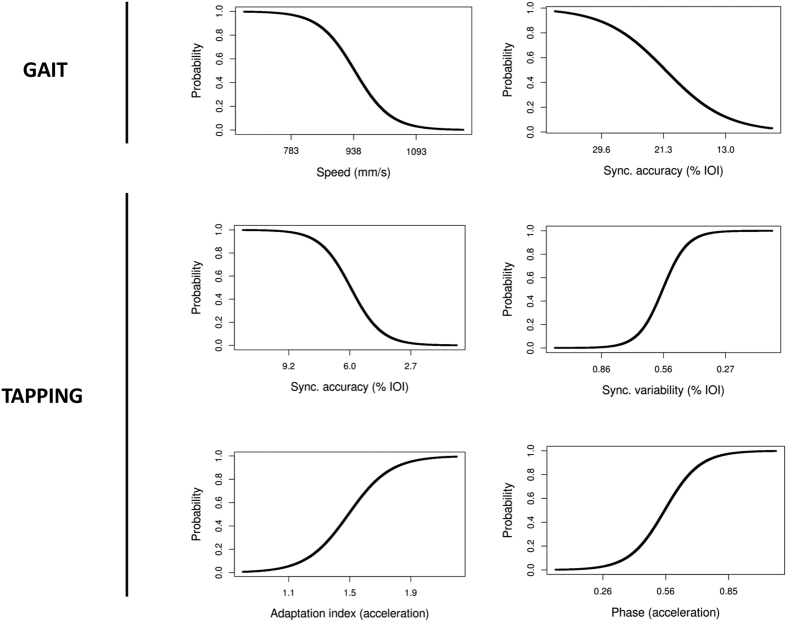
Probability curves extracted for each predictor in the logistic regression model while controlling for all the other predictors. The y-axis is the probability that a patient displays a positive response to MCGT. The three values indicated on the x-axis correspond to the mean values of each variable +/− 1 *SD* calculated from the tested sample of patients.

Prior to analysis all predictor variables were converted to *z*-scores (standard normal distribution) and entered simultaneously into the equation. To facilitate the interpretation of the coefficient in the model, the sign of the z scores has been changed systematically for synchronization accuracy and variability. After this change, larger z values in both cases now indicate an improvement of the performance (i.e., greater accuracy and lower variability). The model provides a highly significant fit as compared to a null model (null −2LL = 11.66, final −2LL = 26.92, *χ*^*2*^ = 30.52, *p* < 0.001; Nagelkerke *R*^*2*^ = 0.73; AIC = 37.32). The coefficients of the model and their significance are reported in Table 3. All predictors attained statistical significance. Probability functions based on the model were computed for each of the predictors after controlling for all other variables, and are presented in Fig. 2. In the Figure, the probability that a participant is going to respond positively to MCGT is indicated. To facilitate the interpretation of the relation between the predictors and the probability of a positive response to the treatment, values for the predictor corresponding to the mean of the tested sample of patients +/− the *SD* are indicated. It can be seen that lower gait speed and, interestingly, lower synchronization accuracy when patients walked with a faster stimulus than their preferential cadence are associated with a greater probability of responding to MCGT. For example, a decrease in overall gait speed by 155 mm/s (i.e., of 1 *SD*) relative to the group average increases by 47% the probability that a patient will show a positive response to the treatment. In contrast, a decrease of synchronization accuracy by 8.3% of the stimulus IOI relative to the group average increases the probability of a positive response by 43%. This finding is confirmed by tapping data which show that lower synchronization accuracy is associated with a greater probability of a positive response to MCGT.

In addition, it can be seen that low synchronization variability in the tapping task, in spite of accuracy, is a condition which favors a positive response to the treatment. For example, it is apparent that a decrease in synchronization variability by 0.3% of the stimulus IOI relative to the group average increases the probability by 49% that a patient positively responds to the MCGT. Finally, the two predictors obtained from the adaptive tapping task, the adaptation index and phase correction when patients reacted to a stimulus acceleration, reveal that a greater response to the tempo change increases the probability of a positive response to the treatment. In particular higher overcorrection (i.e., when the adaptation index >1) and greater phase correction in this tapping task both increase the probability that the patient will positively respond to MCGT. For example, an increase of the adaptation index by 0.4, and by 0.3 for phase correction, relative to the group average, increases by 46% the probability that a patient positively responds to the MCGT.

## Discussion

The goal of the present study was to examine the role of sensorimotor timing skills, tested with gait and tapping tasks, to evaluate the efficacy of RAS in persons with IPD. We observed that a one-month training programme based on RAS, individualized to the patients’ preferred cadence, overall improved spatio-temporal gait parameters, such as speed and stride length in non-cued gait conditions^20^,^61^. This effect was maintained one month after the training programme^20^,^61^. In spite of these beneficial effects at the group level, we note important individual differences between the patients. Half of them showed a positive effect of RAS, ranging from small to large (i.e., an increase of .07 to .32 m/s in gait speed, and from 2.2 to 28.7 cm in stride length), as assessed by a clinically relevant criterion (i.e., increase in gait speed after the training). Other patients either did not show an effect of RAS, or their performance slightly worsened.

This large variability in RAS outcome may raise concerns about the general effectiveness of this type of treatment. Moreover, it points to important questions about the conditions, which foster an optimal response to RAS. One possibility, and one of the motivations of the present study, is that individual differences in sensorimotor skills may be linked to the success of RAS in some patients, and to no effect or worsened responses in others. This possibility was tested by entering patients’ performance on various tapping and gait sensorimotor tasks into a logistic regression model aimed at predicting the probability of a positive response to RAS. The model shows that patients who are most impaired in terms of their gait (i.e., those exhibiting the slowest gait speed), and who show the poorest synchronization accuracy are likely to benefit from RAS. This finding is not totally unexpected. Indeed, the most impaired patients are those who are likely to show the greatest improvement relative to patients who show less impaired gait (i.e., the latter may already perform at ceiling at the pre-test) or better synchronization accuracy. In addition, patients who are the least variable in a synchronized tapping task and the most responsive in adapting their movement timing (tapping) to a change in a pacing sequence (i.e., an acceleration) are those who benefit the most from RAS. An increased tendency to correct movement timing in response to stimulus acceleration (adaptation index >1, overcorrection), accompanied by greater phase correction, favor a positive response to RAS. Thus patients’ flexibility in adapting their walking behavior to a beat frequency departing from their natural cadence may contribute to the success of RAS.

Altogether, these findings provide meaningful insight that RAS is not generally applicable in movement disorders but varies in its outcome success as a function of onset capacities in patients. Note, however, that even if the effects of the aforementioned predictors in the logistic regression model are significant, the sample of patients tested in the present study is relatively small. Moreover, the link highlighted between sensorimotor timing measures and RAS may particularly apply to IPD patients who are mildly to moderately impaired like the current sample. Whether these findings hold for IPD patients with more severe impairments (e.g., H&Y >2), given that advanced IPD is typically accompanied by additional deficits (e.g., cognitive impairments)^3^ is still an open question. Thus, further testing of the logistic model with a larger sample of IPD patients, spanning a wider range of motor impairments, is in order to assess its robustness and generalizability.

What mechanisms are responsible for these positive effects of RAS as a function of individual differences in sensorimotor timing skills? In general, the core process underlying positive effects of RAS may be the stimulus-driven allocation of attention to relevant aspects of gait kinematics, which augments temporal prediction, and thereby facilitates movement planning and initiation^37^,^62^,^63^. Patients showing somehow spared sensorimotor timing skills, namely those who are quite consistent in a sensorimotor task (e.g., showing low variability in tapping to a beat, in spite of being inaccurate), and who can promptly react to a change in stimulation may benefit from the presentation of an external rhythmic cue. RAS would thus provide a regular temporal scaffolding for movement coordination, and facilitate the compensation of patients’ impaired internal timing^28^,^31^.

This compensation process may be mediated by different brain circuitries^10^,^64^. A possibility is that the observed individual differences may reflect patients’ ability to recruit resources from a network, which also subserves timing and motor control^35^,^64^. Disease progression in IPD is associated to the reorganization of networks affording gait and motor control^65^. In particular, the malfunctioning of a cortico-subcortico-cortical circuitry in IPD may be compensated by increased recruitment of mechanisms relying on ‘alternative’ pathways, such as cerebello-thalamo-cortical circuitry, typically only later affected in the disease^34^,^37^,^66^,^67^. This possibility is supported by evidence on enhanced activity of the cerebellar anterior lobule following 1 month of RAS training^36^. It is noteworthy that this same circuitry, and in particular the cerebellum, is also critical in tasks, which afford the coupling of motor movement to a regular external event^68^,^69^,^70^, and in predictive (i.e., feed-forward) movement control^71^. These processes are likely engaged in the tasks that we used to test sensorimotor skills in the present study (i.e., synchronizing footfalls to the beat and paced tapping). Thus, performance in these tasks may be an important indicator of patients’ capacity to engage compensation as a result of RAS. The involvement of compensation by RAS is also compatible with the finding that patients showing the largest error correction during sensorimotor synchronization, a function which is likely to be partly associated with the cerebellum (e.g., phase correction)^72^,^73^, are those who benefit most from RAS.

Another possibility worth considering is that RAS may rely on residual activity in cortico-striatal circuitry that is impaired in IPD. Such activity could afford a minimal amount of beat processing^74^,^75^, which may be sufficient to provide a temporal pacing of movement initiation and execution. Thus, residual temporal processing (e.g., relatively spared extraction of a beat from the acoustic signal) in some of the patients may create optimal conditions for the success of RAS. In sum, whether the effects of RAS linked to individual differences in patient profiles are mediated by a compensation, or by residual activity of impaired timing mechanisms remains an open question that deserves further study.

Altogether, we showed that sensorimotor timing skills in IPD patients, needed when synchronizing steps and hand tapping to an auditory beat, are linked to the success rate of RAS in gait rehabilitation. This finding is relevant to pinpoint the role of general-purpose mechanisms for perceptual and sensorimotor timing^35^,^76^,^77^ in IPD, and to identify the functional link between such mechanisms and training strategies that foster neuronal plasticity. Moreover, these results have important consequences for clinical research. We consider this a first important step towards understanding individual differences in the response to RAS, and in developing individualized training programs for gait disorder in IPD and beyond. By identifying the individual factors, which contribute to the success of RAS, such as initial gait speed, or the performance in sensorimotor timing tasks, the study provides useful insights for IPD patient treatment. Indeed, for some patients it is expected that RAS may have no effect on spatio-temporal parameters of gait, or even potentially negative consequences. Nevertheless, this does not entail that those patients should be discouraged from using RAS. The stimulation may still have beneficial effects above and beyond gait, such as increasing patients’ motivation to walk (for example by using patients’ personal music playlist), which may still increase their quality of life.

## Additional Information

**How to cite this article:** Dalla Bella, S. *et al*. Gait improvement via rhythmic stimulation in Parkinson's disease is linked to rhythmic skills. *Sci. Rep.* **7**, 42005; doi: 10.1038/srep42005 (2017).

**Publisher's note:** Springer Nature remains neutral with regard to jurisdictional claims in published maps and institutional affiliations.

## Supplementary Material

Supplementary Table 1

## Figures and Tables

**Table 1:** Demographic and clinical characteristics for IPD patients and healthy controls.

		Patients		Controls	
		*Mean (SD*)	*n*	*Mean (SD*)	*n*
Demographics					
Females		—	5	—	10
Males		—	9	—	10
Handedness					
	Right	—	14	—	20
Age		66.5 (*7.2*)	14	66.4 (*7.8*)	20
Years of education		14.8 (*2.8*)	14	14.4 (*3.0*)	20
Age at onset		58.5 (*7.1*)	14	—	—
Disease duration		8.0 (*2.8*)	14	—	—
Clinical characteristics					
UPDRS					
	I (Mentation, Behavior and Mood)	2.6 (*1.8*)	14	—	—
	II (Activities of Daily Living)	11.4 (*5.9*)	14	—	—
	III (Motor Examination)	22.8 (*12.7*)	14	—	—
	Total score	36.8 (*19.1*)	14	—	—
Hoehn & Yahr		2.0 (*0.7*)	14	—	—
	0.5	—	1	—	—
	1	—	2	—	—
	2	—	6	—	—
	2.5	—	4	—	—
	3	—	1	—	—
Schwab & England		87.9 (*5.8*)	14	—	—
Medication (mg)					
	L-dopa LED	146.2 (*160.8*)	13	—	—
	Ago LED	241.5 (*204.7*)	13	—	—
	Total LED	360.0 (*270.1*)	14	—	—

**Table 2:** Performance in non-cued and cued gait tasks for IPD patients obtained pre-, post-training and at the follow-up, and for matched controls.

	Controls	Patients			Comparisons (significance)			
		*Pre*	*Post*	*Follow*-*up*	*Pre vs Control*	*Post vs Pre*	*Follow*-*up vs Pre*	
	*Mean (SEM*)	*Mean (SEM*)	*Mean (SEM*)	*Mean (SEM*)	*P*	*P*	*P*	
***Gait*** - ***Non-Cued conditions***								
Cadence (step/min)	100.5 (*1.8*)	106.3 (*2.3*)	108.0 (*2.2*)	109.5 (*2.2*)	p < 0.05	p = 0.05	p < 0.05	
Speed (mm/sec)	964.4 (*25.9*)	898.9 (*48.1*)	952.7 (*37.0*)	961.5 (*39.3*)	p = 0.09	p = 0.05	p < 0.05	
Stride length (mm)	1152.0 (*22.3*)	1011.7 (*44.8*)	1057.5 (*37.8*)	1053.7 (*38.7*)	p < 0.05	p < 0.05	p < 0.05	
Stride length variability	10.4 (*0.7*)	10.8 (*0.9*)	10.4 (*0.9*)	10.8 (*0.6*)	p = 0.45	p = 0.27	p = 0.43	
Stride time (sec)	1.2 (*0.02*)	1.1 (*0.03*)	1.1 (*0.02*)	1.1 (*0.02*)	p < 0.05	p < 0.05	p < 0.05	
Stride time variability	.0048 (*0.0003*)	.0048 (*0.0008*)	.0036 (*0.0003*)	.0040 (*0.0005*)	p = 0.19	p < 0.05	p = 0.13	
***Gait*** - ***Cued conditions***								
−10%								
Inter-step interval (ms)	627.7 (*14.0*)	610.3 (*16.4*)	576.3 (*15.9*)	572.3 (*14.6*)	p = 0.26	p < 0.05	p < 0.01	
Sync. accuracy (% of IOI)	10.8 (*8.9*)	22.7 (*3.1*)	22.4 (*2.0*)	24.9 (*2.8*)	p < 0.01	p = 0.32	p = 0.34	
Sync. variability (% of IOI)	1.3 (*0.3*)	2.0 (*0.5*)	2.2 (*0.6*)	2.8 (*1.0*)	p = 0.1	p = 0.27	p = 0.42	
+10%								
Inter-step interval (ms)	535.7 (*11.0*)	518.6 (*13.4*)	513.4 (*11.4*)	500.4 (*12.4*)	p = 0.19	p = 0.31	p < 0.05	
Sync. accuracy (% of IOI)	18.0 (*2.3*)	19.1 (*3.4*)	23.3 (*1.6*)	21.4 (*1.8*)	p = 0.48	p = 0.12	p = 0.26	
Sync. variability (% of IOI)	2.3 (*0.5*)	1.3 (*0.4*)	3.5 (*0.6*)	3.5 (*0.6*)	p = 0.18	p < 0.05	p < 0.01	

**Table 3:** Logistic regression model predicting patients’ response to MCGT (0 = no positive response, 1 = positive response) based on their performance in cued gait tasks and tapping tasks.

Predictor		*B*	SE (*B*)	exp(*B*)	Wald test	*P*
	Intercept	0.06	0.53	NA	0.12	n.s.
**Gait**						
	Speed	−3.50	1.48	0.03	−2.36	<0.05
	Sync. Accuracy (+10%)	−2.04	1.03	0.13	−1.98	<0.05
**Tapping**						
	Sync. Accuracy	−3.97	1.69	0.02	−2.35	<0.05
	Sync. Variability	5.08	2.17	160.38	2.34	<0.05
	Adapt. Index (acceler.)	2.91	1.34	18.31	2.17	<0.05
	Phase (acceler.)	3.58	1.31	35.85	2.73	<0.01

NA = not applicable.
