# Supplementary material for: Gait improvement via rhythmic stimulation in Parkinson’s disease is linked to rhythmic skills
Source: Sci Rep. 2017 Feb 24;7:42005. doi: 10.1038/srep42005 (PMC5324039; doi:10.1038/srep42005)
Supplement: Supplementary Table 1 [file srep42005-s1.pdf]

SUPPLEMENTARY INFORMATION

Gait improvement via rhythmic stimulation in Parkinson's disease is linked to rhythmic skills

Simone Dalla Bella, Charles-Etienne Benoit, Nicolas Farrugia, Peter Keller, Hellmuth Obrig, Stefan Mainka & Sonja A. Kotz

Supplementary Table 1: Performance in paced tapping and adaptive tapping tasks for IPD patients obtained pre-, post-training and at the follow-up, and for matched controls.

	Controls	Patients			Comparisons (significance)		
		Pre	*Post*	*Follow-up*	*Pre vs Control*	*Post vs Pre*	*Follow-up vs Pre*
Paced tapping	*Mean (SEM)*	*Mean (SEM)*	*Mean (SEM)*	*Mean (SEM)*	*P*	*P*	*P*
**450 ms**							
**Inter-step interval (ms)**	450.3 *(.3)*	449.8 *(.2)*	449.8 *(.2)*	450.0 *(.2)*	p = .22	p = .36	p = .26
**Sync. accuracy (% of IOI)**	4.9 *(.6)*	6.8 *(1.3)*	5.4 *(.5)*	7.0 *(1.1)*	p = .11	p = .29	p = .43
**Sync. variability (% of IOI)**	.59 *(.09)*	.54 *(.04)*	.55 *(.04)*	.52 *(.05)*	p = .37	p = .48	p = .21
**600 ms**							
**Inter-step interval (ms)**	599.7 *(.2)*	599.8 *(.2)*	603.7 *(3.6)*	599.8 *(.1)*	p = .38	p = .10	p = .35
**Sync. accuracy (% of IOI)**	6.8 *(1.1)*	6.3 *(.8)*	6.8 *(1.2)*	5.8 *(1.1)*	p = .28	p = .50	p = .33
**Sync. variability (% of IOI)**	.52 *(.04)*	.52 *(.02)*	.60 *(.1)*	.47 *(.03)*	p = .11	p = .33	p < .05
**750 ms**							
**Inter-step interval (ms)**	749.8 *(.2)*	750.1 *(.5)*	749.8 *(.3)*	749.7 *(.3)*	p = .30	p = .38	p = .18
**Sync. accuracy (% of IOI)**	3.9 *(.5)*	5.4 *(.9)*	5.7 *(1.4)*	4.3 *(.8)*	p = .09	p = .20	p < .05
**Sync. variability (% of IOI)**	.47 *(.03)*	.55 *(.04)*	.76 *(.3)*	.54 *(.06)*	p = .08	p = .25	p = .29
Adaptive tapping							
**Deceleration**							
**Adaptation index**	1.3 *(.1)*	1.2 *(.2)*	1.2 *(.1)*	1.3 *(.2)*	p = .27	p = .40	p = .31
**Phase**	.51 *(.07)*	.71 *(.1)*	.53 *(.1)*	.59 *(.1)*	p = .20	p = .09	p = .18
**Period**	.92 *(.1)*	.78 *(.1)*	.99 *(.1)*	1.1 *(.1)*	p = .16	p = .34	p = .05
**Acceleration**							
**Adaptation index**	1.5 *(.1)*	1.6 *(.08)*	1.4 *(.1)*	1.5 *(.1)*	p = .29	p = .07	p = .23
**Phase**	.50 *(.07)*	.58 *(.07)*	.59 *(.07)*	.50 *(.09)*	p = .22	p = .36	p = .28
**Period**	1.2 *(.1)*	.96 *(.09)*	.85 *(.09)*	1.1 *(.1)*	p = .19	p = .22	p = .27
